# The Bacterium of Diphtheria

**Published:** 1881-04

**Authors:** Ch. Talamon


THE BACTERIUM OF DIPHTHERIA. A series of experiments made by Ch. Talamon, *ex-interne des hôpitaux*, and reported to the Anatomical Society, January, 1881.

Most of my experiments have been made with false membranes from patients in the service of Dr. Bergeron, Sainte-Eugénie Hospital. Thanks to his favors and those of his interne, Dr. Suss, I have been able to gather a certain number of false membranes detached from the tonsils, from the trachea, or expelled through the canula, after tracheotomy. I will simply mention here such cases as gave positive results, which are eight in number, all cases of infectious sore-throat, true diphtheria, either confined to the pharynx or extended to the larynx and trachea. There were two cases complicated with croup, and in both the false membrane cultivated had been taken from the trachea, and gave the same organism as false membranes from the tonsils. In one case of diphtheria, the first in which I discovered the bacterium now presented to you, I was able to follow the disease from its start till recovery. The patient was a man 20 years old, sleeping in the same room with a friend attacked with a malignant form of diphtheria, and he had a sore throat from the day on which the latter died. The day following, he came to the Hôtel-Dieu, and we were able to discover false membranes, greenish-gray, on both tonsils, and a marked swelling of the lymphatics. Every day I repeatedly cultivated these false membranes, and during the whole course of the disease, which lasted ten days, I constantly reproduced the same microscopical organism. After the membranes had disappeared, mucus from the back of the throat repeatedly cultivated gave no result.

This is the only case in which I was able to follow, day after day, the course of the disease. In the rest of the cases I took false membranes once from each. Here is the organism produced by the cultivation in each of the eight cases. It does not resemble the *zygodesmus fuscus* of Letzerich, which has never been seen since, not even by Letzerich himself, nor the *tilletia diphtherica*, which received the claims of the German author after he had given up the zygodesmus, nor the *microsporon* of Klebs, which bred but one micrococcus. It is a fungus easily seen and recognized, whose evolution I was able to observe and study in every detail, either in the large partitioned cultivations or in damp cells.

After its evolution is completed, it appears under the shape of mycelia and characteristic spores. These mycelia sometimes appear as long tubes, partitioned from distance to distance, specially refracting the light, and generally very clear; they are from 2 to 4 and 5 thousandths of millimeters wide (m .000002 to .000005). When the fluids are well conditioned they stretch extremely, at times branch off, and these divisions are themselves peculiar; they form by their slightly incurved branches a figure best compared to a lyre or a tuning fork. At other times, the mycelia do not stretch in that way; although multiplying, so as to rapidly cover the surface of the liquid of cultivation, they remain short, take on odd shapes, the most common of which resembles a crutch; there is always present then a multitude of straight cylinders, 4 or 5 thousandths of millimeters wide by 15, 20 or 40 thousandths long (m .000004 by .000015 or .00004).

There are two kinds of spores—round or oval—which may be considered the spores of germination, and rectangular spores which represent the last phase of growth of the fungus, and which we will call *conidia* (*conidies*). These give the species its particular character; their shape is rectangular and their size very liable to vary; their width varies from m .0J0001 to .000008 and more; their length from m .000005 to .000015. Sometimes they are isolated, other times they are in groups of 2 or 3; often they form beads of 10, 12 or 15 individuals, or a small chain in zig-zag. Homogeneous at first, they soon become filled with small round bodies, very brilliant, the size of common micrococci, which appear to me as the true germ of the fungus.

The round or oval spores are what forms the mycelium by stretching; they look like bright specks m .000003 to .00005 in diameter, in the middle of granular matter, spread in sheets of greater or lesser extent, which represent what is called *zooglœa*. These spores stretch one of their poles so as to form a tube m .000002 to .000004 in diameter, which branches as above stated. When the lengthening of the mycelium is first begun, the space with its prolongation represents a tadpole.

In experimenting with bacteria, it is not sufficient to isolate, by cultivation, the organism supposed to be the first cause of the disease. It is not sufficient that the organism should kill the animal experimented upon. One must—and here is the difficulty—reproduce, if possible, in an animal the disease referred to the organism, or at least prove, through a cultivation of the various organs, that the organism inoculated, and not septicæmia are accountable for the animal's death. Now, in spite of what some German authors affirm, no one as yet has been able to reproduce diphtheria. Letzerich and Klebs pretended having obtained it in rabbits: they killed rabbits indeed; one after injecting into them the *zygodesmus fuscus* during a first period, and the *tilletia* during the second; the other after inoculating them with the *microsporon;* but neither reproduced diphtheria, for neither of them reproduced false membranes, and the rapidity with which their rabbits died leads one to think that death resulted from nothing else than septicæmia.

I inoculated the bacteria first described, under the mucous membrane of the mouth and nose of, or gave it to eat to, six rabbits, two guinea-pigs, four frogs, a cock and four pigeons. The six rabbits died in the course of six, eight, ten, or eighteen days. The first died after six days with enormous swelling of the neck, to be compared with the œdema of diphtheria. It consisted in a serous infiltration of the cellular tissue, and a cultivation of the serum has reproduced the bacterium with characteristic *conidia.* The rabbit whose death occurred eighteen days after the ingestion of liquids containing the bacteria, had a double fibrinous pleuritis with effusion; the effused liquid as well as the false membranes reproduced through cultivation the inoculated organism. In all the rabbits, indeed, I found, sometimes with the help of the microscope alone, at other times through cultivation, the fungus always present in the serum of the peritoneal cavity, often in that of the pericardium, and also in the kidneys; but blood taken from the heart never reproduced the organism. The liquid would generally remain clear in that case, or common bacteria would be found.

Of the two guinea-pigs one alone died. I let three frogs swallow flocculi of mycelia two or three times; they died 8, 10 and 11 days after the last ingestion. They were, so to speak, stuffed with the bacteria. Specially in the subcutaneous lymphatis and in the lymph duct, the organism had abundantly multiplied. Here again the peritoneal fluid has reproduced the bacteria in cultivations, while the blood taken from a heart yet beating gave no result; in another case bacteria were produced. Two of these frogs were rutting; the female's abdomen was full of spawn; the male's testicles were the size of small beans and full of sperm. The sperm and the spawn gave the diphtheritic bacterium under cultivation, in such a manner that had fecundation been possible, the tadpoles might have had at birth the germs of diphtheria.

The most marked lesion in frogs is in the stomach. The mucous membrane was covered with a thick layer of yellowish viscous mucus, consisting, when formed under the microscope, of epithelial cells, of granulations and drops of fat, of a multitude of micrococci, and of a certain number of rectangular *conidia*. The fourth frog had, besides, an intense inflammation of all the coats of the stomach, whose peritoneal aspect was covered with fine and recent false membranes.

I let the cock swallow some flocculi of mycelium. After eight days, as he did not seem to become sick, I desired to cool his temperature, and I left him with his legs in cold water during twenty-four hours, following the course of Pasteur for the refrigeration of hens sick with anthrax. His temperature effectually fell from 42° to 38° C. Unhappily the board on which the cock was retained slipped into the water and he drowned. On making the autopsy, I found the mucous membrane of the crop covered with a multitude of white specks the size of a lentil; these under the microscope were found to consist of epithelium filled with granulations, globules of fat, micrococci, and a great many rectangular spores.

Finally, I succeeded in reproducing false diphtheritic membranes in the four pigeons. After scratching deeply with the blade of a bistoury the surface of the mucous membrane, then smearing the solution over the abrasions, I noticed after twenty-four hours, a thick membrane coating both sides of the mouth, the tongue, *velum palati*, and the back of the throat; this false membrane was whitish yellow, and formed, like the false membranes of the pharynx and of the tonsils in man, of epithelial cells, of fat, of cocci, and of bacteria; very few rectangular *conidia* were present, but under cultivation this membrane always reproduced the peculiar organism. I saw no fibrin. Two of the pigeons died after three days; one of them had false membranes around the opening of the larynx also, and its trachea was filled with a thick mucus whose cultivation reproduced the bacterium. The peritoneal and pericardial fluids and the kidneys reproduced it also under cultivation. But, as in the case of rabbits and frogs, the blood taken from the heart did not contain it, and the flasks sowed with it remained clear. The third pigeon recovered after eight days of sickness; the false membranes came off and he got well. The fourth is yet under observation. These various experiments present peculiarities on which I will insist later. My aim in this paper has been confined to prove that I had isolated a microscopical organism which has better claims than the *tilletia* of Letzerich, and the *microsporon* of Klebs, to be acknowledged as the cause of diphtheria, since it reproduces false membranes which these authors never produced.

---

"HUNTER took pupils, as formerly, into his house, as was then the custom all over England, and spent all the time he could snatch from his practice in the study of comparative anatomy and natural history, and in making preparations for his museum. His habit was to rise at four o'clock in the morning, to spend from four to five hours in his dissecting-rooms, and then to step into his carriage to make his daily rounds among his patients. He had no fondness for surgical practice or consultations, and attended to business only because it afforded him the means of purchasing objects of curiosity or of natural history, saying, as he laid aside his scalpel and forceps, 'Well, Lynn,' a pupil and an intimate friend, 'I must go and earn that d—d guinea, or I shall be sure to want it to-morrow.'"—*Dr. S. D. Gross.*

